# Supporting Parents of Young Children With Type 1 Diabetes Through Telehealth: Randomized Controlled Trial of the Reducing Emotional Distress for Childhood Hypoglycemia in Parents Intervention

**DOI:** 10.2196/86616

**Published:** 2026-04-30

**Authors:** Susana R Patton, Nicole Kahhan, Holly O'Donnell, David D Williams, Mark Clements, Kimberly Driscoll

**Affiliations:** 1 Center for Healthcare Delivery Science, Nemours Children's Clinic, 807 Children's Way, Jacksonville, FL, 32207, United States, 1 9046973595, 1 9046973425; 2 Division of Psychology, Nemours Children's Clinic, Jacksonville, FL, United States; 3 Barbara Davis Center for Diabetes, University of Colorado Anschutz Medical Campus, Aurora, CO, United States; 4 Health Services and Outcomes Research, Children's Mercy Kansas City, Kansas City, MO, United States; 5 Division of Pediatric Endocrinology, Children's Mercy Kansas City, Kansas City, MO, United States; 6 Department of Clinical and Health Psychology, University of Florida Health Science Center, Gainesville, FL, United States

**Keywords:** pediatric diabetes, type 1 diabetes, hypoglycemia fear, parenting, caregivers, digital health, psychosocial outcomes

## Abstract

**Background:**

Parents of young children with type 1 diabetes (T1D) are vulnerable to experiencing fear of hypoglycemia (FH), an emotional condition that includes persistent and intense worry about hypoglycemia and/or use of unhealthful behaviors to avoid hypoglycemia. Despite greater uptake of continuous glucose monitors (CGMs) and automated insulin delivery systems, FH remains prevalent and under-addressed in parents of young children. As such, we developed Reducing Emotional Distress for Childhood Hypoglycemia in Parents (REDCHiP), a video-based telehealth intervention designed to reduce FH in parents by providing T1D education and teaching parents how to apply evidence-based strategies from cognitive behavioral therapy and behavioral parent training in their child’s daily T1D care.

**Objective:**

This study aimed to compare the REDCHiP intervention to a novel attention control condition (ATTN) to better isolate treatment effects for REDCHiP based on parents’ FH and diabetes distress.

**Methods:**

This was a multisite randomized controlled trial. We enrolled 197 families and randomized 183 to either REDCHiP or ATTN. Both REDCHiP and ATTN parents completed 10 video-based telehealth sessions. Our primary outcome was changes in parental FH; secondary outcomes included changes in parental diabetes distress and children’s glycated hemoglobin A_1c_ (HbA_1c_). We used a series of mixed-effects models and logistic regression to evaluate treatment effects.

**Results:**

Parents in REDCHiP and ATTN attended >95% of sessions with high treatment fidelity. FH and diabetes distress decreased significantly over time in both REDCHiP and ATTN. Treatment-slope effects slightly favored REDCHiP but were not statistically significant for FH (*P=*.09) or distress *(P=*.06). However, parents receiving REDCHiP were significantly less likely to report clinically elevated diabetes distress over time compared to ATTN *(P=*.02). Child HbA_1c_ showed a small, nonsignificant reduction over time *(P=*.06). Parents with elevated depressive symptoms consistently reported higher FH and distress across all time points.

**Conclusions:**

REDCHiP demonstrated high feasibility, acceptability, and potential clinical relevance in reducing diabetes distress among parents of young children with T1D. While overall treatment effects were modest, use of an attention control condition represents a meaningful advancement in trial rigor for pediatric behavioral interventions. Future adaptations of REDCHiP may enhance its impact, particularly for parents experiencing comorbid depressive symptoms.

## Introduction

Young children (<8 years old) living with type 1 diabetes (T1D) are particularly vulnerable to hypoglycemia due to heightened insulin sensitivity [1,2], unpredictable eating and exercise patterns [3], and challenges in recognizing and self-reporting symptoms of hypoglycemia [4]. Consequently, severe hypoglycemia occurs at nearly twice the rate in young children compared to older children and adolescents [5]. Parents, who bear primary responsibility for managing their young child’s T1D regimen, are vulnerable to experiencing fear of hypoglycemia (FH) [4,6]. FH is a multisymptom emotional condition characterized by persistent and intense worry about the occurrence of hypoglycemia and/or reliance on unhealthful behaviors to avoid hypoglycemia (eg, treating normal glucose levels and underdosing insulin) [4]. FH affects up to 60% of parents of young children with T1D [4,7] and relates to lower quality of life [8,9], higher parenting stress [10], higher diabetes-related distress, and burnout, perhaps related to parents’ experiencing a constant state of vigilance for hypoglycemic events [11].

Despite the high prevalence and negative impact of FH, there are few evidence-based interventions specifically designed to address this emotional challenge in parents of young children with T1D. To fill this gap, our team developed Reducing Emotional Distress for Childhood Hypoglycemia in Parents (REDCHiP) [12,13], a video-based telehealth intervention that merges principles of cognitive behavioral therapy and behavioral parent training with T1D education to help parents reduce their feelings of FH and their reliance on unhealthful hypoglycemia avoidance behaviors.

REDCHiP conceptualizes parents’ FH as a specific phobia, which is readily treatable with cognitive-behavioral therapy [14,15]. Specifically, parents learn to identify unhelpful thoughts, feelings, and behaviors related to managing their child’s T1D. Parents build a personal fear hierarchy of specific anxiety-provoking T1D-related situations and identify current maladaptive coping strategies that they desire to change. Parents then learn cognitive and behavioral strategies to help manage their fear while challenging themselves to gradually navigate fearful T1D-related situations or events differently (eg, response prevention). Finally, through imaginary and in vivo exposures, parents practice using the strategies they have learned to reduce feelings of FH [12].

Both individual and group treatment delivery appear effective when targeting anxiety [16] and providing behavioral parent training [17]. In the context of T1D education, group-based delivery is common [18]. REDCHiP combines individual and group treatment delivery so that parents can both receive personalized care during individual sessions and benefit from social support and the shared lived experiences of other parents of young children with T1D in the group sessions. In recent years, widespread uptake of video-based telehealth has transformed the delivery of pediatric behavioral interventions [19]. These platforms offer unique advantages for busy families, including less travel burden [20], flexible scheduling [21,22], and the ability for families to receive care in the home environment [21]. By designing REDCHiP for delivery via telehealth, we aimed to enhance its accessibility among parents of young children with T1D across diverse geographic locations.

To develop REDCHiP, we followed the Obesity-Related Behavioral Intervention Trials (ORBIT) model for behavioral treatment development [23]. This systematic approach to treatment development has multiple phases, each comprised of different preliminary studies designed to yield the incremental data necessary to increase the likelihood that the final treatment can influence a clinically meaningful target. In an earlier ORBIT Phase 2a study, we completed a waitlist-controlled pilot trial of REDCHiP [12] that included 36 families. We had high retention (86%), high session attendance (94%), and high parental satisfaction [24]. Moreover, our between-group comparisons demonstrated a significant reduction in FH for REDCHiP parents compared to waitlist parents, and once all parents had received REDCHiP, we found significant reductions in parents’ FH and diabetes distress from pre- to posttreatment [12].

Thus, as a next step, we conducted an ORBIT Phase 2b study to better determine the source of our REDCHiP treatment effects [23]. Specifically, in a multisite randomized clinical trial (RCT), we compared our REDCHiP intervention to an attention control condition (ATTN). Our primary hypothesis was that parents randomized to REDCHiP would report greater reductions in FH compared to parents randomized to ATTN. However, we also explored changes in parents’ diabetes distress as a secondary outcome, given its association with parents’ FH and our pilot data, and we explored any reductions in young children’s glycated hemoglobin A_1c_ (HbA_1c_) following parental participation in REDCHiP or ATTN. If parents were purposefully engaging in behaviors to avoid the occurrence of hypoglycemia due to FH, we expected that HbA_1c_ could decrease among young children whose parents received REDCHiP versus ATTN. Here, we report the outcomes of our ORBIT Phase 2b trial of the REDCHiP intervention.

## Methods

### Study Design

This multisite RCT enrolled parents of young children with T1D from all regions of the United States through one of 3 recruitment sites located in the Southeast, Midwest, or Mountain regions of the United States. Our primary outcome was change in parental FH. Our secondary outcomes were change in parental diabetes distress and children’s HbA_1c_. We measured outcomes pretreatment, immediately post treatment, and 3-month post treatment, with the last assessment point intended to explore any maintenance effects.

### Procedures

We recruited participants from 2019 to 2023; however, to comply with COVID-19 restrictions, we halted recruitment from March to July 2020. Additionally, due to slow recruitment, in August 2022, we expanded our efforts and launched a media campaign through the Type 1 Diabetes Exchange to advertise for the trial nationally. Our inclusion criteria were parents (including legal guardians) of young children with T1D, aged 2‐6.99 years old, ≥6 months post diagnosis, and using intensive insulin therapy. We excluded parents if their child had a comorbid chronic condition (eg, renal disease), their child was allergic to the adhesive or skin preparation used for continuous glucose monitors (CGMs), their child was on conventional insulin therapy, or parents did not identify as English speaking. We did not restrict families based on the child’s HbA_1c_, though we required young children to be at least 6 months post diagnosis to allow for their HbA_1c_ to begin to stabilize following insulin initiation. We targeted parents who self-identified as primarily responsible for their child’s daily T1D management to complete the treatment sessions and study questionnaires. However, parents who identified as a secondary caregiver for daily T1D management were also welcome to attend the treatment sessions, and multiple caregivers from the same family were able to participate together if preferred, though only one caregiver was included in our analyses.

We used both in-clinic and remote recruitment procedures. We screened all families who expressed an interest in participating in the trial for eligibility. After obtaining parent-informed consent, we randomized families 1:1 to either our treatment (REDCHiP) or ATTN. Families then completed visit 1, which included REDCap (Research Electronic Data Capture) [25] questionnaires and a measurement of child HbA_1c_ via a validated home kit, which was analyzed in a central lab. Families completed visit 2 at the end of the treatment phase and visit 3 approximately 3 months later. Visits 2 and 3 repeated the same study procedures as visit 1.

We compensated families for completing the study visits (US $35 per study visit). Parents received this incentive via a reloadable debit card (Clincard). We also gifted children a toy, valued at up to US $15, at each study visit as a thank you for completing the home HbA_1c_ kit.

### Study Conditions

#### REDCHiP

Parents randomized to REDCHiP participated in 10 video-based telehealth sessions completed over a 12-week period [13]. There was a combination of individual and group sessions. Initially, parents attended 3 individual sessions and 7 group sessions. However, due to COVID-19 and scheduling challenges, we modified the format to 7 individual and 3 group sessions without changing content. Individual and group sessions typically lasted between 30 and 60 minutes each.

REDCHiP leaders included licensed psychologists, supervised psychology trainees, and a certified diabetes educator. They completed structured training in REDCHiP content and delivery, shadowed experienced leaders, and co-led sessions before leading sessions independently. They also received ongoing supervision from one of the designers of REDCHiP during intervention delivery to promote fidelity to the manual and consistent application of cognitive-behavioral strategies. For individual sessions, parents worked one-on-one with a REDCHiP leader, while group sessions brought together up to 5 parents and 1‐2 trained REDCHiP leaders. We digitally recorded sessions and reviewed about one-third to code for treatment fidelity.

#### ATTN

Parents randomized to ATTN completed 10 video-based telehealth sessions over 12 weeks, matching REDCHiP in dose and format [13]. Initially, ATTN included 3 individual and 7 group telehealth sessions, but we later modified the schedule to 7 individual and 3 group sessions to remain consistent with REDCHiP. Individual and group sessions lasted 30‐60 minutes.

ATTN leaders included supervised psychology trainees and clinical trials assistants. They completed structured training in ATTN content and delivery, shadowed experienced leaders, and co-led sessions before leading independently. During the sessions, parents learned content relevant to all parents of young children, such as important developmental milestones, child health and safety, school readiness, early childhood literacy, and positive parenting strategies. Leaders redirected any T1D-related questions to the child’s T1D care team. We digitally recorded sessions and reviewed about one-third to code for treatment fidelity.

### Measures

Families randomized to REDCHiP or ATTN completed the same online questionnaires, and young children completed a validated home HbA_1c_ kit. We previously published a description of our trial battery [13]. For this report, we focus on describing our sample characteristics and examining treatment effects based on changes in parent FH and diabetes distress and child HbA_1c_.

#### Family and Children’s T1D History

In visit 1, parents completed a study-specific questionnaire to describe themselves (age, sex, relationship to the child, race and ethnicity, marital status, and highest grade completed) and their child (age, sex, race and ethnicity, date of T1D diagnosis, T1D treatment regimen, and history of severe hypoglycemia).

#### Hypoglycemia Fear

At all study visits, parents completed the 22-item Hypoglycemia Fear Survey-Parents of Young Children (HFS-PYC) [6], which measures FH based on worries and the frequency of hypoglycemic avoidance behaviors. Parents respond to items using a 5-point Likert scale (1=Never to 5=Very Often), with higher scores indicating greater fear. In line with published scoring recommendations, we removed 4 items with poor performance for young children on intensive insulin therapy, resulting in a modified initial total score (range=22‐110). To enhance interpretation, we rescored the HFS-PYC to fit a 0‐100 scale by calculating the item mean score and multiplying by 20.

#### Diabetes Distress

At all study visits, parents completed the 18-item Problem Areas in Diabetes-Parent Revised (PAID-PR) [26], which is a validated measure of parent-reported diabetes distress. Parents respond to items using a 5-point Likert scale (0=Not a problem-4=Serious problem). The PAID-PR scoring uses a 0‐100 scale with higher scores indicating greater distress. Additionally, a total score ≥56 may indicate the presence of significantly elevated diabetes distress [27].

#### Depressive Symptoms

At all study visits, parents completed the 20-item Center for Epidemiological Studies Depression Scale-Revised (CESD-R) [28,29], which is a measure of depressive symptoms over the past 2 weeks. Parents respond to items using a 5-point Likert scale (0=Not at all-4=Nearly every day). Based on normative data, a total score ≥16 suggests elevated depressive symptoms [29]. We dichotomized parent depressive symptoms, categorizing CESD-R scores ≥16 as “elevated depressive symptoms” and scores <16 as “non-elevated symptoms.” We included the presence of elevated depressive symptoms as a covariate in all models because of its known association to FH and distress in the extant literature [30,31].

#### Child HbA_1c_

At all study visits, children completed a validated home HbA_1c_ kit [32] to obtain a proxy measure of children’s average glycemia using a finger-prick blood sample. We analyzed the samples in a single laboratory using automated high-performance liquid chromatography with measurement methods reliable to Diabetes Control and Complications Trial standards (reference range 4.0%‐6.0%; Tosoh 2.2; Tosoh Corporation) [33].

### Sample Size and Power

We conservatively based our original power analysis on the assumption of completing 40 groups (20 REDCHiP and 20 ATTN) with an average size of 3.6 parents (40×3.6=144, or a final sample of n=144 parents). Based on this sample size, a standardized effect size of 0.4 SDs, and an intraclass correlation of 0.10, we projected an estimated power of 85%. We originally estimated an attrition rate of 20%, thereby setting our target sample size at 180 families. However, during the trial years affected by COVID-19, we experienced a higher rate of attrition. Thus, to account for this higher attrition rate, we over-recruited for the trial; our final sample comprised 197 families.

### Statistical Analyses

To compare families randomized to REDCHiP or ATTN based on sociodemographic characteristics collected at visit 1, we used independent 2-tailed *t* tests, chi-square tests, or 2-sample Wilcoxon rank-sum tests for nonnormally distributed variables. Because preliminary analyses indicated correlations between parent depressive symptoms and both FH and distress, we elected to include the presence of elevated parental depressive symptoms as a covariate in all our models to better isolate treatment effects. To examine reductions in parents’ FH (primary outcome), we ran a mixed effects model with participant as a random effect and fixed effects for randomization (REDCHiP or ATTN), visit (slope of the respective outcome over the study timeline), and the interaction of randomization by study timeline. For diabetes distress (secondary outcome), we repeated the mixed effects model when analyzing distress as a continuous variable. We conducted a logistic regression to analyze distress dichotomously (elevated vs not elevated) as a sensitivity analysis to examine whether parents were less likely to report clinically elevated distress over time based on an established threshold, using the same random effect structure and covariate. In exploratory analyses, we examined reductions in child HbA_1c_ by repeating the mixed effects model. We conducted all analyses in StataNow/SE 18.5 (StataCorp LLC). Because our hypotheses were directional and prespecified, we applied one-sided *P* values to test for changes in the expected direction. We did not adjust for multiplicity because we designed the trial as an ORBIT Phase 2b study exploring treatment effects rather than providing definitive efficacy estimates [23]. Consistent with CONSORT (Consolidated Standards of Reporting Trials; Checklist 1) reporting guidelines [34], we report estimates of effect sizes for observed treatment-slope trends based on the methods described by Feingold [35] to aid interpretation of the magnitude of change; they should not be interpreted as evidence of statistical significance.

### Ethical Considerations

We operated this multisite RCT under a single institutional review board (IRB) agreement, with Children’s Mercy Hospital-Kansas City as the IRB of record. We used IRB-approved procedures to obtain electronic documentation of parent informed consent before enrolling parents into the trial. All children were younger than 7 years; therefore, we did not require separate child assent. We registered the trial on ClinicalTrials.gov (NCT3914547) before proceeding with any trial activities. We collected and managed study data using secure, password-protected systems and deidentified all data prior to analysis. Participation involved minimal risk, and parents could skip questionnaire items or withdraw from the study at any time without penalty. We compensated participants for their time.

## Results

### Sample Characteristics

**Figure 1.**
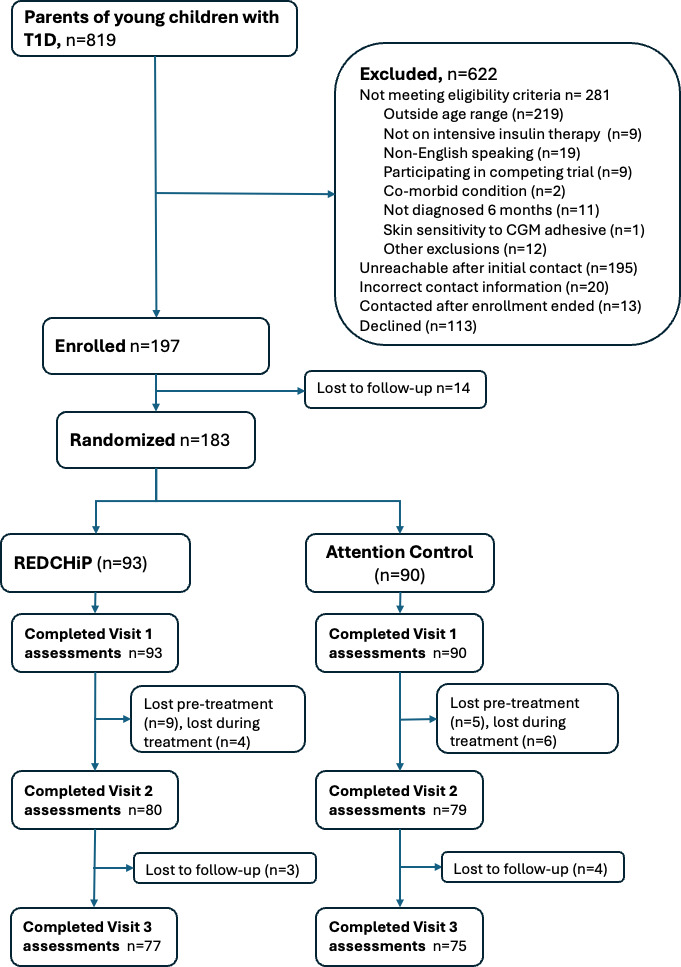
CONSORT (Consolidated Standards of Reporting Trials) diagram. REDCHiP: Reducing Emotional Distress for Childhood Hypoglycemia in Parents.

We enrolled 197 families of young children with T1D and randomized 183 families (n=93 to REDCHiP and n=90 to ATTN). At visit 1, we collected parent and child demographics. Young children had a mean age of 4.4 (SD 1.2) years, a mean HbA_1c_ of 7.54% (SD 1.15), and a mean time since diagnosis of 1.67 (SD 1.15) years. Children were 55% (100/183) male. Parents had a mean age of 36.3 (SD 5.9) years and 93% (170/183) self-identified as the child’s mother. Table 1 displays sample characteristics and comparisons by group. The CONSORT table details our recruitment and retention across the seven-and-a-half-month trial (Figure 1). Of the total enrolled, 77% (152/197) of families completed all study visits.

**Table 1.** Sample characteristics.

Demographics	Overall (n=183)		REDCHiP^a^ (n=93)	ATTN^b^ (n=90)
Child characteristics				
Age (years), visit 1, mean (SD)	4.4 (1.2)		4.5 (1.2)	4.3 (1.2)
Sex (male), n (%)	100 (55)		51 (55)	49 (54)
Parent characteristics				
Visit 1, n	177		90	87
Age (years), mean (SD)	36.3 (5.9)		36.7 (5.3)	35.9 (6.6)
Sex (female), n (%)	170 (93)		84 (90)	86 (96)
Marital status (partnered), n (%)	169 (92)		90 (97)	79 (88)
Highest parental education (college graduate and above), n (%)	113 (62)		61 (66)	52 (58)
Family race and ethnicity				
Non-Hispanic White, n (%)	147 (80)		80 (86)	67 (74)
Non-Hispanic Black, n (%)	5 (3)		1 (1)	4 (4)
Hispanic or Latinx, n (%)	18 (10)		3 (3)	15 (17)
Asian, n (%)	2 (1)		2 (2)	0 (0)
Native American, n (%)	1 (<1)		0 (0)	1 (1)
More than one race, n (%)	10 (5)		7 (8)	3 (3)
Clinical characteristics				
HbA_1c_^c^ (visit 1), n	178		88	—^d^
HbA_1c_ (%), mean (SD)	7.54 (1.15)		7.42 (1.02)	7.65 (1.26)
Since diagnosis (at visit 1), n	182		—	89
Time since diagnosis (years), at visit 1, mean (SD)	1.67 (1.15)		1.55 (1.00)	1.78 (1.28)
CGM^e^ use, n (%)	151 (83)		78 (84)	73 (81)
AID^f^ use, n (%)	93 (51)		45 (48)	48 (53)

a REDCHiP: Reducing Emotional Distress for Childhood Hypoglycemia in Parents.

b ATTN: attention control condition.

c HbA_1c_: hemoglobin A_1c_.

d Not applicable.

e CGM: continuous glucose monitor.

f AID: automated insulin delivery.

### Intervention Characteristics

Overall, parents randomized to REDCHiP attended 95.2% of sessions, and parents randomized to ATTN attended 95.9% of sessions. While there was no difference in the mean length of group sessions for the REDCHiP and ATTN arms (*P*=.22), there was a difference in the mean length of individual sessions for the REDCHiP and ATTN arms (*P=*.003). Specifically, individual sessions in the REDCHiP arm were on average 7 minutes longer in duration than individual sessions in the ATTN arm (mean 39.6, SD 4.2 vs mean 32.7, SD .7 minutes for REDCHiP and ATTN, respectively). We coded treatment fidelity for 170 out of 517 (33%) of ATTN sessions and 199 out of 532 (37%) of REDCHiP sessions using structured, session-specific checklists. Overall, both arms achieved a high treatment fidelity rate (85/100, 85% for ATTN and 89/100, 89% for REDCHiP).

### Survey Outcomes

Table 2 describes outcomes for parent FH, diabetes distress, and the percentage of parents with clinically elevated depressive symptoms for the entire sample and within each treatment arm. Across both arms, parent FH, diabetes distress, and the percentage of parents with clinically elevated depressive symptoms decreased from visits 1 to 3. However, at all study visits, parents with clinically elevated depressive symptoms reported higher FH and distress than parents without clinically elevated depressive symptoms (*P=*.001; Table 3).

**Table 2.** Survey outcomes at each time point by group.

Overall	REDCHiP^a^			ATTN^b^		
	Visit 1	Visit 2	Visit 3	Visit 1	Visit 2	Visit 3
Hypoglycemia fear, mean (SD)	55.44 (13.42)	49.43 (10.95)	46.89 (9.46)	57.43 (14.38)	53.30 (13.24)	51.75 (12.02)
Diabetes distress, mean (SD)	43.00 (19.70)	29.57 (16.67)	28.01 (15.29)	46.14 (20.90)	39.05 (22.05)	34.70 (20.16)
Clinically elevated depressive symptoms, n (%)	40 (43)	22 (28)	18 (23)	37 (41)	23 (29)	15 (20)

a REDCHiP: Reducing Emotional Distress for Childhood Hypoglycemia in Parents.

b ATTN: attention control condition.

**Table 3.** Hypoglycemia fear and diabetes distress over time by presence of elevated depressive symptoms.

*insertMeasure	Parents with elevated depressive symptoms	Parents without elevated depressive symptoms	*P* value
Visit 1, mean (SD)			
Hypoglycemia fear	61.74 (13.92)	52.56 (12.59)	.001
Diabetes distress	57.18 (17.21)	35.36 (17.26)	.001
Visit 2, mean (SD)			
Hypoglycemia fear	57.49 (11.90)	48.93 (11.57)	.001
Diabetes distress	47.31 (20.20)	29.13 (17.55)	.001
Visit 3, mean (SD)			
Hypoglycemia fear	55.48 (11.36)	47.57 (10.36)	.001
Diabetes distress	49.16 (16.23)	26.35 (15.32)	.001

### Mixed Effects Models

In a series of mixed effects models, we examined treatment effects for parents’ FH and diabetes distress, with randomization (REDCHiP or ATTN), visit (slope of the respective outcome over study timeline), interaction of randomization by study timeline, and presence of elevated depressive symptoms as covariates. In our first model, results revealed that parents’ FH decreased over time for both treatment arms (coefficient=−2.57; 95% CI −3.81 to −1.33; *P=*.001), with a treatment-slope effect that slightly favored the REDCHiP group, though it was not significant (coefficient=−1.16; 95% CI −2.88 to 0.57; *P*=.09). Similarly, in our second model, results revealed a decrease in parents’ diabetes distress over time for both treatment arms (coefficient=−3.93; 95% CI −5.63 to −2.23; *P*=.001), with a nonsignificant treatment slope effect (coefficient=−1.87; 95% CI −4.23 to 0.50; *P*=.06) that slightly favored the REDCHiP group. Although the interaction terms were not statistically significant, we calculated effect sizes to describe the magnitude of observed trends in accordance with CONSORT recommendations [34]. For parents’ FH, the effect size for REDCHiP was Feingold d=0.18, and for diabetes distress, the effect size for REDCHiP was Feingold d=0.21.

### Logistic Regression

We also examined treatment effects for parents dichotomized as having either clinically elevated or not elevated diabetes distress, with randomization (REDCHiP or ATTN), visit (slope of the respective outcome over the study timeline), interaction of randomization by study timeline, and presence of elevated depressive symptoms as covariates. There was no time effect (coefficient=−0.25; 95% CI −0.61 to −0.11; *P*=.08). However, we observed a significant treatment-slope effect (coefficient=−0.70; 95% CI −1.35 to −0.05; *P*=.02), suggesting that, over time, REDCHiP parents were less likely to endorse clinically elevated diabetes distress.

### Glycemic Outcomes

In mixed effects models, we explored treatment effects for children’s HbA_1c_, with randomization (REDCHiP or ATTN), visit (slope of the respective outcome over study timeline), interaction of randomization by study timeline, and presence of elevated depressive symptoms as covariates. Results revealed a small but nonsignificant reduction in child HbA_1c_ over time for both treatment arms (coefficient=−0.05; 95% CI −0.12 to 0.03; *P*=.11), with a nonsignificant treatment-slope effect for children of parents in the REDCHiP group (coefficient=0.04; 95% CI −0.06 to 0.15; *P*=.21).

## Discussion

### Overview

Our aim was to examine the source of treatment effects for REDCHiP, a telehealth-delivered intervention to reduce FH in parents of young children with T1D, by conducting an RCT. We recruited a large sample of parents from across the United States and randomized them to receive either REDCHiP or an attention control treatment (ATTN). We hypothesized parents receiving REDCHiP would report greater reductions in FH and diabetes distress compared to parents who were randomized to ATTN. We also explored whether children experienced an HbA_1c_ reduction based on parents’ exposure to REDCHiP or ATTN.

### Primary Results

Contrary to our primary hypothesis, our results suggest that all participating parents experienced significant reductions in FH with treatment-slope effects that slightly favored REDCHiP parents and were nonsignificant (*P*=.09). When we examined parents’ diabetes distress as a secondary outcome, our model with diabetes distress as a continuous variable showed a similar time and treatment slope effect (*P*=.06). However, the model analyzing diabetes distress as a dichotomous variable identified a significant treatment-slope effect for REDCHiP. Specifically, we found that parents receiving REDCHiP were less likely to report clinically elevated diabetes distress over time than parents receiving ATTN (*P*=.02). Our results also suggest a small and nonsignificant decrease in children’s HbA_1c_ over time, regardless of intervention group.

Although both REDCHiP and ATTN were associated with reductions in FH and diabetes distress, the reasons for improvement in the ATTN group remain unclear. ATTN sessions did not include content on emotional regulation or stress management, and there is limited evidence that general support groups reduce anxiety or depressive symptoms in adults [36]. One possibility is that nonspecific factors, such as social connection during sessions (especially surrounding the COVID-19 pandemic), contributed to improvements, though this is only speculative and would require further dismantling studies to confirm. Importantly, we designed ATTN as a comparator versus its own scalable intervention. Furthermore, it is unknown whether a longer follow-up post treatment would reveal greater divergence between groups. In a context where virtually no interventions exist to address FH in parents of young children with T1D, we believe the observed reductions, even if nonsignificantly different by condition, represent an important step forward in the field.

While there is an emerging trend in testing new behavioral interventions uniquely tailored to parents of young children with T1D, the literature remains small compared to parents of older children with T1D [37]. As such, we believe this trial adds valuable insight into how to support parents of young children with T1D in managing symptoms of FH and diabetes distress. Moreover, because this is the first behavioral intervention trial among parents of young children with T1D to use an attention control arm, we assert that it marks a notable improvement in trial design rigor directed at families of young children with T1D [38,39].

Our trial recorded a very high attendance rate (~95% of sessions attended) for parents randomized to REDCHiP and ATTN. We think the telehealth format of our treatments was likely the main contributor to our high session attendance. Parents could participate from home, thereby eliminating typical barriers to traditional, in-person behavioral health sessions (eg, transportation, childcare, and time off from work) [19,21,22]. However, it is possible another contributor to our high attendance rate may be parents’ keen interest in receiving support for the emotional and behavioral challenges of managing T1D in their young child. Indeed, our telehealth format allowed us to recruit nationally, potentially reaching families who may not have access to behavioral health care through their local diabetes center [40]. Thus, it appears REDCHiP’s telehealth format helped enhance feasibility and engagement in this trial and may provide preliminary evidence to support its broader dissemination and integration into pediatric diabetes care through existing telehealth infrastructure.

Though we did not find support for our main hypothesis, our trial builds on previous research that suggests that parents of young children with T1D may be particularly vulnerable to FH [3,6]. Parents in this trial reported baseline levels of FH that were comparable to levels reported in published studies [6]. We think this is notable in the context of our sample’s high rate of personal CGM (151/183, 83%) and automated insulin delivery (AID; 93/183, 51%) use. Within the larger literature, the results are mixed as to whether CGM and AID use are associated with lower FH in parents of young children [11,41-43]. Thus, our data may further support the notion that some parents require treatment specifically focused on their fear to experience any improvements [36]. We also think it is notable that REDCHiP parents experienced an absolute mean reduction in FH (−8.22) that was comparable to our previous REDCHiP pilot trial (−9.02) [12]. This may suggest that REDCHiP is still an effective treatment in helping parents to learn the skills to manage FH, even though the reduction was only slightly and non-significantly greater than among ATTN parents.

Our trial examined reductions in parents’ diabetes distress using 2 approaches, as a continuous variable and as a dichotomous variable based on clinically elevated versus nonelevated diabetes distress. We examined distress as a continuous variable to provide a precise estimate of treatment effects and calculate effect sizes. We conducted a sensitivity analysis using the dichotomous measure to assess clinical significance, whether parents moved from elevated to nonelevated distress, a threshold clinics would likely use to guide referral and treatment decisions. Results suggested that REDCHiP had a small effect on parents’ diabetes distress when analyzed continuously, with an absolute mean reduction (−12.88) comparable to our pilot trial (−11.11) [12]. This suggests REDCHiP helped parents manage diabetes distress, even if the treatment-specific effect was attenuated by the ATTN group. However, when analyzed dichotomously, exposure to REDCHiP was associated with a lower occurrence of elevated distress over time compared to ATTN, suggesting REDCHiP may help parents achieve clinically meaningful reductions in diabetes distress. This finding may be particularly relevant for clinical practice.

It is important to recognize the impact of a parent report of elevated depressive symptoms on FH and diabetes distress. At every assessment point and regardless of treatment group, parents with elevated depressive symptoms reported significantly higher FH and diabetes distress than parents without depressive symptoms. Unfortunately, we conducted this trial during the COVID-19 pandemic, a time when depressive symptoms were on the rise and parents of children vulnerable to COVID-19 were more likely to report depressive symptoms than adults prepandemic [44]. While not formally recorded, many parents in our trial described limiting social behaviors or not enrolling children in childcare or preschool. Many parents reported challenges in balancing working from home with parenting secondary to the COVID-19 pandemic. Moreover, some parents described additional concerns specific to COVID-19 and its potential added risk in youth with chronic illness. In our earlier REDCHiP pilot, we did not observe a change in parents’ depressive symptoms after exposure to the intervention [12]. We believe this may be because REDCHiP focuses specifically on changing parents’ unhelpful thoughts and behaviors related to fear. Nevertheless, the data from this trial suggest that parents with elevated depressive symptoms may require a different treatment approach. Therefore, a future direction could be to adapt REDCHiP to incorporate strategies specifically targeting depressive symptoms, such as behavioral activation [45], to better support parents experiencing depressive symptoms and FH.

While our trial demonstrated a small but nonsignificant reduction in child HbA_1c_ related to parent exposure to either REDCHiP or ATTN, this outcome may be expected. In our REDCHiP pilot, we only observed a small change in HbA_1c_ among young children who started the trial with an HbA_1c_ that was above the clinical target [12]. In the current sample, nearly one-third of young children had a baseline HbA_1c_ <7%. This suggests the possibility that we may have encountered a floor effect; there was simply not enough variability in young children’s HbA_1c_ to detect a meaningful change. In the future, it may be helpful to use young children’s CGM data to explore any glycemic changes related to their parents’ exposure to REDCHiP or stratify enrollment by baseline child HbA_1c_ to better detect glycemic effects.

### Limitations

Our use of a large national sample may enhance the generalizability of our results, though as a limitation we acknowledge that our sample was majority non-Hispanic White and that future research should explore the efficacy of REDCHiP in a more racially and ethnically diverse sample. Related, we acknowledge a limitation in that our trial primarily recruited mothers versus fathers and other caregivers. Because less is known about FH in fathers and other caregivers of young children with T1D [4], it is important that future research explore these other caregivers’ experience with FH and response to treatment. We acknowledge a limitation in that we did not restrict our sample to only include families of children with an above-target HbA_1c_. We made this decision based on past data showing moderately high levels of FH even among parents of young children with an HbA_1c_ at or below their target [6]. Nonetheless, to see any child glycemic effects related to parents’ exposure to REDCHiP, it may be important for future trials to limit enrollment to families of young children with an above-target HbA_1c_. Because this trial included multiple outcomes and 1-sided *P* values without adjustment for multiplicity, we acknowledge the risk of type 1 error. We made these analytic decisions to prioritize clinical relevance and to explore treatment effects in this Phase 2b trial. Nevertheless, future studies should confirm these findings using more conservative approaches. As important strengths of the trial, we highlight the high rate of CGM and AID use among young children, our use of a central laboratory to analyze children’s HbA_1c_, our acceptable retention rate, our high attendance rate (~95% of sessions), our relatively low rate of data missingness (<10%) [46], and our rigorous trial design that included an attention control condition [38] and an assessment of treatment fidelity.

### Conclusions

In summary, even with uptake of CGM and AID in young children with T1D, some parents continue to experience FH and diabetes distress and may require specialized treatment. REDCHiP is a telehealth-delivered intervention to reduce FH in parents of young children with T1D. Though our trial shows treatment-slope effects that only slightly and nonsignificantly favored REDCHiP parents with respect to FH and diabetes distress, we remain optimistic with respect to REDCHiP’s potential treatment impact. We found that parents’ exposure to REDCHiP was associated with a lower occurrence of elevated diabetes distress over time than ATTN. Also, we observed that parents exposed to REDCHiP reported high treatment satisfaction. REDCHiP aligns with broader trends in pediatric digital behavioral health with its remote delivery, personalization, and integration of evidence-based strategies from cognitive behavioral therapy and behavioral parent training into parents’ daily T1D care [19]. Moreover, because of its telehealth format, it is possible REDCHiP could be integrated into pediatric diabetes care as a first-line treatment, leaving more intensive in-person treatment for families with more complex needs [40]. Future refinements and adaptations of REDCHiP will aim to increase its treatment effect, especially for parents experiencing comorbid depressive symptoms and young children with elevated HbA_1c_. We also intend to explore ways to tailor REDCHiP for other caregiver populations, including fathers, grandparents, and teachers. Finally, future adaptations of REDCHiP will explore how to incorporate other digital health delivery methods, such as asynchronous content for self-paced learning and mobile app integration to track and reinforce skill learning to further promote its scalability and implementation.

## Supplementary material

10.2196/86616 Checklist 1 CONSORT checklist for eHealth trials.

## Abbreviations:

AID: automated insulin delivery
ATTN: attention control condition
CESD-R: Center for Epidemiologic Studies Depression Scale-Revised
CGM: continuous glucose monitor
CONSORT: Consolidated Standards of Reporting Trials
FH: fear of hypoglycemia
HbA_1c_: hemoglobin A_1c_
HFS-PYC: Hypoglycemia Fear Survey-Parents of Young Children
IRB: institutional review board
ORBIT: Obesity-Related Behavioral Intervention Trials
PAID-PR: Problem Areas in Diabetes-Parent Revised
RCT: randomized clinical trial
REDCap: Research Electronic Data Capture
REDCHiP: Reducing Emotional Distress for Childhood Hypoglycemia in Parents
T1D: type 1 diabetes
